# Pore-Forming Proteins: From Pore Assembly to Structure by Quantitative Single-Molecule Imaging

**DOI:** 10.3390/ijms24054528

**Published:** 2023-02-25

**Authors:** Eleonora Margheritis, Shirin Kappelhoff, Katia Cosentino

**Affiliations:** Department of Biology/Chemistry and Center for Cellular Nanoanalytics (CellNanOs), University of Osnabrück, 49076 Osnabrück, Germany

**Keywords:** pore-forming proteins, protein complex stoichiometry, single-molecule fluorescence microscopy, super-resolution microscopy, pore functionality

## Abstract

Pore-forming proteins (PFPs) play a central role in many biological processes related to infection, immunity, cancer, and neurodegeneration. A common feature of PFPs is their ability to form pores that disrupt the membrane permeability barrier and ion homeostasis and generally induce cell death. Some PFPs are part of the genetically encoded machinery of eukaryotic cells that are activated against infection by pathogens or in physiological programs to carry out regulated cell death. PFPs organize into supramolecular transmembrane complexes that perforate membranes through a multistep process involving membrane insertion, protein oligomerization, and finally pore formation. However, the exact mechanism of pore formation varies from PFP to PFP, resulting in different pore structures with different functionalities. Here, we review recent insights into the molecular mechanisms by which PFPs permeabilize membranes and recent methodological advances in their characterization in artificial and cellular membranes. In particular, we focus on single-molecule imaging techniques as powerful tools to unravel the molecular mechanistic details of pore assembly that are often obscured by ensemble measurements, and to determine pore structure and functionality. Uncovering the mechanistic elements of pore formation is critical for understanding the physiological role of PFPs and developing therapeutic approaches.

## 1. Introduction

Pore-forming proteins (PFPs) are a big class of proteins sharing the ability to form pores in a wide variety of target membranes, ranging from bacteria to humans [1,2]. Within the PFP family, pore-forming toxins (PFTs) are produced as part of the attack or defense machinery of different organisms and act exogenously, permeabilizing the membrane of target cells [3,4]. PFTs directly kill target cells as pore formation disrupts the plasma membrane permeability barrier required for maintaining cell homeostasis. Other PFPs act endogenously in the cell, like in the case of proteins involved in death signaling programs, namely apoptosis, pyroptosis, and necroptosis [5,6]. PFPs involved in regulated cell death are activated following genetically encoded and tightly regulated signaling pathways initiated by the cell in response to the detection of dangerous or death signals. In this case, membrane pores allow the intra- or extra-cellular transfer of ions, proteins, and other constituents to activate downstream cellular signaling events, often leading to inflammatory responses and cell suicide. Regulated cell death PFPs are therefore related to key biological processes including development, metabolism, infection, and immunity [7,8]. 

The general mechanism of pore formation is considered an ancient process that involves several steps common to all PFPs. First, proteins, which are generally soluble, bind to membrane receptors (including specific lipids), thus ensuring target specificity. Upon binding, PFPs usually undergo conformational changes that lead to membrane insertion, protein oligomerization, and, in the final step, pore formation. However, PFPs greatly vary in their levels of protein organization, from their primary sequence to their quaternary structure in the pore complex, and this impacts their ability to bind membranes, oligomerize into a certain number of units, and form pores of different sizes and with different functionalities. Elucidating the molecular details of this complex sequence of events for a given PFP requires appropriate investigative tools that allow for dissecting the conformational changes proteins undergo during complex assembly in the membrane. Additionally, the spatial-temporal heterogeneity of pore formation has to be taken into account. For example, multiple pores at a cellular membrane may be at different stages of their process of formation at a given time, or intermediate structures may already be able to permeabilize the membrane and thus play a functional role. 

In this review, we present an overview of PFPs, with a special focus on those acting in regulated cell death. We will describe the process of pore formation of representative PFPs, highlighting how critical aspects, such as differences in the mechanism of assembly and stoichiometry as well as the cooperation between proteins and lipids, affect the final pore structure and its functionality to the passage of signaling molecules. We will then discuss the challenges of investigating the mechanisms of action of PFPs and how recent technological developments in biophysics have contributed to the unveiling of the molecular mechanisms of pore formation. We will specifically focus on single-molecule techniques as powerful tools to dissect the structural heterogeneity of pore formation, both in cells and in model membrane systems. 

## 2. Pore-Forming Proteins (PFPs)

PFPs have in common the ability to permeabilize cell membranes, which often leads to cell death. Cell death can be non-regulated if it is triggered by exogenous PFPs, such as PFTs, or regulated (regulated cell death—RCD) if it is executed by endogenous PFPs activated by suicide mechanisms of the cell [6].

In this section, we introduce some relevant examples of PFTs and regulated cell death PFPs, describing their mechanisms of activation and assembly, as well as the properties of the membrane pore. We will refer to α-PFPs and β-PFPs, based on the secondary structure they adopt in the membrane-integrated state, corresponding to α-helical vs. β-barrel pores (Table 1) [9].

### 2.1. Pore-Forming Proteins in Non-Regulated Cell Death

PFTs, a large subgroup of PFPs, function by perforating the membranes of target cells, leading to ion imbalance, water influx, and eventually cell death [4,38]. Relevant PFT families include the membrane attack complex/perforin super-family (MACPF), cholesterol-dependent cytolysins (CDCs), actinoporins, aerolysins, cytolysin A, colicins, and hemolysins (Table 1) [39]. 

PFTs are produced by both prokaryotic and eukaryotic organisms for manifold purposes. For example, MACPF proteins, such as perforin and the membrane attack complex MAC, have been integrated by eukaryotic cells as part of their defense mechanism and represent relevant components of the immune systems to target and kill bacteria as well as infected cells [40]. Perforin is a four-domain glycoprotein produced and secreted, together with the serine protease granzymes, by killer lymphocytes, including cytotoxic T lymphocytes (CTLs) and natural killer (NK) cells. This death mechanism is well conserved and includes the formation of an immunological synapse between the lymphocyte and the target cell. Perforins and granzymes are packaged into cytotoxic secretory granules (called “lytic granules”) that are trafficked and fused to the presynaptic membrane, thus allowing their release into the synaptic cleft. At the postsynaptic membrane, perforins form large transmembrane pores that enable the secretion of granzymes into the target cell where they trigger cell death, eliminating cancerous or infected cells. The pore of perforin allows the translocation of a monomer of Granzyme B or a dimer of Granzyme A [41]. The perforin-granzyme pathway has relevant implications for immune homeostasis and for developing immune-based therapies to treat cancer, infection, and autoimmunity [3]. 

Other PFTs are instead virulence factors produced by many pathogenic bacteria and represent an offensive mechanism against the host [39]. Amongst them, CDCs are a β-PFT family of microbial virulence factors mostly produced by several species of Gram-positive bacteria belonging to the *Bacillus*, *Clostridium*, *Streptococcus*, and *Listeria* genera and that mediate severe infectious diseases in humans. Depending on the pathogen, CDCs trigger infection in different ways. The CDC family member Listeriolysin O (LLO) is produced by the bacterium *Listeria monocytogenes*, the pathogen responsible for causing listeriosis. During *Listeria* invasion of host cells, LLO pores are responsible for both bacterial internalization after host plasma membrane damage and disruption of the phagosome membrane, which allow bacteria to escape into the cytosol and rapidly multiply intracellularly [17,42,43,44]. Moreover, LLO acts to suppress the immune system of the host by impairing SUMOylation of critical proteins involved in infection responses [45,46]. Pneumolysin (PLY) is another CDC family protein and is produced by the bacterium *Streptococcus pneumoniae*, the main cause of respiratory infections and pneumonia worldwide, as well as infectious complications such as cardiac dysfunction and meningitis. PLY acts at different levels of pneumococcal infection. Initially, it aids the bacteria during colonization by facilitating adherence to the host. Subsequently, during the invasion, it is involved in bacterial penetration and phagosomal escape by causing direct damage to host cell membranes through the formation of pores of 26 nm in diameter [47]. During the process of infection, it interferes with the host´s immune response by activating the classical complement pathway and leading to a decrease in the availability of complement components, thereby reducing the clearance of *S. pneumoniae* by neutrophils [48,49]. In the early stage of infection, at sub-lytic concentrations, PLY promotes cell survival by stimulating cell repair mechanisms and cytoskeleton reorganization and reducing inflammation. At lytic concentrations, it fosters dysregulation of cellular homeostasis, cellular pro-inflammatory signaling pathways, mitochondrial damage, and cell death [50].

### 2.2. Pore-Forming Proteins in Regulated Cell Death

Membrane permeabilization is also a common feature in the execution of regulated cell death that involves tightly regulated signaling cascades and molecularly defined effectors. Here, we present the main relevant protein families involved in pore formation in regulated cell death. Specifically, we focus on the Bcl-2 family, whose members control intrinsic apoptosis by regulating the permeabilization of the mitochondrial outer membrane (MOM), on members of the Gasdermin protein family that control plasma membrane (PM) permeabilization in pyroptosis, and, finally, on mixed lineage kinase domain-like (MLKL) protein, the pore-forming executor of necroptosis.

#### 2.2.1. Membrane Pore Formation in Apoptosis: Bax and Bak

Apoptosis is a form of programmed cell death that occurs during development and aging as a homeostatic mechanism to maintain cell population balance in tissues, as well as for the correct functioning of the immune system and as a defense mechanism in immune responses. Apoptosis downregulation is the main cause of tumorigenesis, while its upregulation induces autoimmune and neurodegenerative diseases [41]. Apoptosis involves the activation of cysteine proteases, called caspases, and a complex cascade of events following an extrinsic or an intrinsic pathway. The extrinsic pathway is activated when external ligands such as the tumor necrosis factor–α (TNFα) bind to death receptors. The consequent oligomerization of the receptor triggers the recruitment and the activation of caspase 8 and caspase 10, which in turn activates the effector caspases 3 and 7, leading to apoptosis. The intrinsic or mitochondrial apoptotic pathway is activated by internal stimuli such as DNA damage, glucocorticoids, endoplasmic reticulum (ER) stress, hypoxia, and metabolic stress. It is controlled by the Bcl-2 protein family and involves mitochondrial outer membrane permeabilization (MOMP). The Bcl-2 family comprises more than 20 members, classified into anti-apoptotic and pro-apoptotic subgroups considering their function in apoptosis and the number of Bcl-2 homology (BH) domains they contain. The proapoptotic members of the family include the MOMP executors Bax and Bak. In healthy cells, Bak is mainly located at the MOM while Bax is mainly localized into the cytoplasm. After apoptosis induction, Bax and Bak accumulate at distinct foci on the mitochondrial surface where they undergo conformational changes, assemble, and oligomerize, executing MOMP. MOMP facilitates the release of pro-apoptotic proteins such as cytochrome c (Cyt C) into the cytosol. Cyt C interacts with the apoptotic-protease-activating factor1 (APAF1), triggering the apoptosome assembly and the activation of caspase 9, which, in turn, activates caspase 3 and 7, leading to apoptosis [51].

In its inactive form, Bax, the best-studied member of the family, presents a globular structure with nine α-helices with a central hydrophobic hairpin (α-5) surrounded by a bundle of amphipathic helices exposed to the aqueous environment. Upon apoptosis, activation of Bax (and Bak) is initiated by interaction with BH3-only activators that trigger Bax (and Bak) conformational changes, promoting an extended membrane-inserted structure. After membrane insertion of the α9 helix of Bax, Bax (and Bak) assembly at the membrane proceeds via the collapse of the helical hairpin α5-α6 to facilitate the formation of dimeric units [52,53]. Bax and Bak dimers further assemble into oligomers with an even number of molecules to form protein-lipid pores that reach hundreds of nanometers in size over time [32,33,34,54,55]. Fast organization of Bak into small structures seems to precede Bax and enhance Bax recruitment and kinetics of assembly [54].

#### 2.2.2. Membrane Pore Formation in Pyroptosis: The GSDM Family

Pyroptosis is a highly inflammatory, lytic form of regulated cell death initiated in response to pathogen cell invasion or detection of dangerous cytosolic perturbations. Pyroptosis plays an important role in immune protection against intracellular infection by eliminating the compromised cell, thus removing the pathogen’s protective niche and simultaneously eliciting an inflammatory response. However, its dysregulation is responsible for inflammatory disorders [56]. 

At the signaling level, pyroptosis can follow different pathways, all culminating in the activation of members of the Gasdermin (GSDMs) pore-forming family. The GSDM family is composed of six members in humans (GSDMA-GSDMB-GSDMC-GSDMD-DFNAr or GSDME-DFNB59 or PJVK), most of which have been shown to have pore-forming activity. Amongst them, GSDME and GSDMD are so far the best-characterized [8,57]. In the canonical and non-canonical pathways, pyroptosis is induced by the activation of the inflammatory caspase-1 and caspases 4\5 (11 in mice), respectively, and culminates in the downstream activation of the Gasdermin (GSDMs) family member GSDMD. Upon caspase activation, the p30 N-terminal pore-forming domain (NTD) of GSDMD is cleaved from its regulatory p20 C-terminal inhibitory domain (CTD). Subsequently, the NTD translocates to the PM and oligomerizes to form large membrane pores that allow the release of cellular contents, including the pro-inflammatory cytokines interleukin-1β and -18 and drive swelling and membrane rupture. It has also been proposed that GSDMD is able to form pores on bacterial membranes to kill intracellular invading bacteria [57,58]. 

#### 2.2.3. Membrane Pore Formation in Necroptosis: MLKL

Necroptosis is a form of regulated cell death involved in development, immune response to viral infection, and inflammatory injury. Deregulation of necroptosis has been associated with pathological conditions such as cancer and neurodegenerative and inflammatory diseases [59,60]. Necroptosis is induced by Toll-like receptors, death receptors, interferons, and T-cell receptors [61,62,63].

After necroptosis induction, two kinases, receptor-interacting serine/threonine-protein kinase 1 (RIPK1) and receptor-interacting protein kinase 3 (RIPK3), are sequentially activated. Upon activation, RIPK1 and RIPK3 recruit MLKL to form the necrosome signaling complex. In particular, MLKL is activated by phosphorylation by RIPK3 at residues T357 and S358, ultimately leading to necrosis through PM disruption and cell lysis. MLKL comprises an N-terminal four-helix bundle domain (4 HB), and a C-terminal pseudo-kinase domain (psK) connected by a flexible bridge formed by a two-helix linker. The active N-terminal four-helix bundle domain (4 HB) is considered the pore-forming domain and it is suggested to oligomerize and make pores of 4 nm in diameter [37,64,65].

## 3. Mechanisms of Pore Formation 

**Figure 1:**
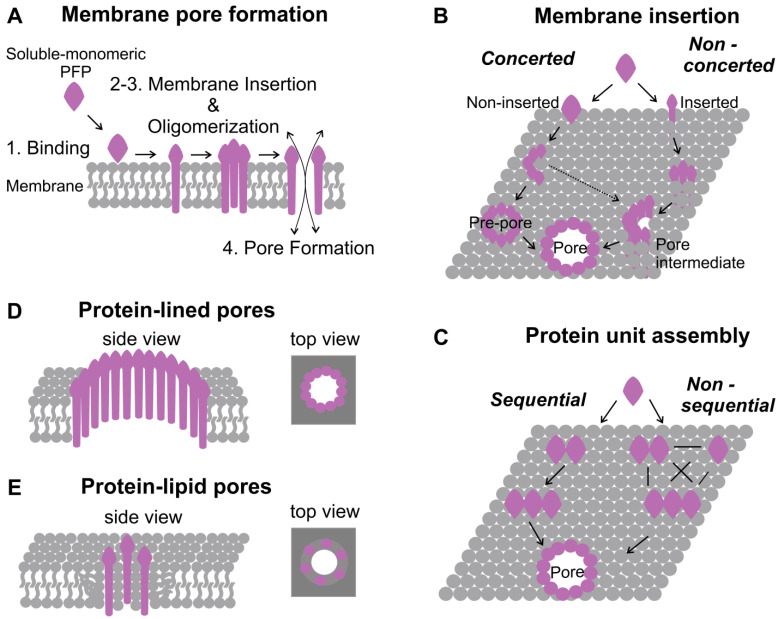
Mechanism of pore formation by PFPs. (**A**) Model of membrane pore formation that involves an initial membrane-protein binding, followed by protein conformational changes that trigger membrane insertion and oligomerization and the final pore formation (details are in the text). (**B**) Mechanism of membrane insertion: concerted vs. non-concerted. In the concerted model of insertion, oligomerization takes place in the first step, leading to the formation of a “pre-pore” above the membrane, while membrane insertion occurs consequently by concerted conformational changes in all protein units. Intermediate non-inserted structures can also insert generating pores (dashed line)In the non-concerted model, protein monomers bind to the membrane, and membrane insertion occurs before or during oligomerization. In this case, intermediate structures can already permeabilize the membrane. (**C**) Mechanism of protein assembly: sequential vs. non-sequential. Protein oligomerization can occur via the sequential addition of units of fixed stoichiometry or by the random (non-sequential) addition of units of different stoichiometries. (**D**,**E**) Schematic representation of protein-lined (**D**) and protein-lipid (**E**) pores. Protein-lined pores are exclusively formed by proteins, while the protein-lipid pore structure derives from the cooperation of both protein and lipids. Lipids are represented in gray and proteins in pink.

The general mechanism of pore formation consists of four steps, including targeting of the activated protein to the membrane, membrane insertion, oligomerization, and pore assembly (Figure 1A). Despite these steps being common for all PFPs, the exact sequence of events may differ for different classes of PFPs. In this section, we describe in detail each step and provide examples of how differences in the mechanisms of pore formation lead to different pore structures [1,2,66,67,68].

### 3.1. Protein Recruitment to the Membrane and Protein-Lipid Cooperation 

In their inactive state, PFPs usually are present as monomers in a water or membrane-embedded state. Activation of PFPs can occur in several ways, including protein cleavage, changes in the protein environment, or interaction with particular sugars, proteins, or lipids.

Following or concomitant to activation, PFPs are recruited to the membrane by specific lipids or proteins that act as receptors. The CDC family member Intermidilysin (ILY), a PFT secreted by the Gram-positive bacterium *Streptococcus intermedius* that specifically lyses human erythrocytes, uses the glycosyl-phosphatidylinositol (GPI)-anchored membrane protein huCD59 as a cellular receptor. Thanks to the specificity of ILY for huCD59, based on its recognition of the C8α- and C9-binding domain of huCD59, this interaction not only allows ILY membrane recruitment but also provides specificity for human cells [69]. Other members of the CDC family and the members of the actinoporin family target membranes via interaction with specific lipids [68]. In particular, CDCs bind cholesterol-rich membranes. Of the four functional domains comprising CDCs, the C-terminal domain 4 is the membrane-sensing domain, recognizing cholesterol via a highly conserved undecapeptide sequence, known as the tryptophan-rich region, and a threonine-leucine amino acid pair. Once domain 4 successfully binds to cholesterol in the membrane, the CDC monomers oligomerize into a pre-pore intermediate, which then inserts into the membrane [70]. The pore-forming activity of PFT actinoporins, such as Equinatoxin II (EqtII) and Frageceatoxin C (FraC), isolated from the sea anemones *Actinia equina* and *Actinia fragacea*, respectively, is boosted by the presence of Sphingomyelin (SM) in the target membrane, which acts as a receptor for these toxins, enhancing their binding through interaction with multiple binding sites [71,72,73,74,75]. The structure of the monomeric state of actinoporins consists of a β-sandwich of two β-sheets, each one connected by loops. This core is flanked by two α-helices, one of which is exposed at the N-terminus. Binding of actinoporins to the SM-containing membranes is ensured by a region rich in aromatic residues, and by the translocation of the N-terminal α-helix to the plasma membrane. Finally, pore assembly occurs on the surface of the membrane, through oligomerization of the N-terminal α-helices that are inserted into the membrane, with concomitant lipid membrane reorganization [12].

Lipids can also play an opposite role and prevent pore formation. This is the case for perforins, where phosphatidylserine (PS)-rich membranes allow the binding of the protein but decrease pore formation. In this case, perforin aggregates into dysfunctional plaques, causing perforin dysfunction [76].

Lipid specificity of PFPs also allows discriminating the targeting of specific organelles within eukaryotic cells. The Bcl-2 family member Bax, responsible for executing MOMP, is recruited to the mitochondrial outer membrane via interaction with cardiolipin (CL), a lipid solely found on mitochondria or bacterial cell membranes [77,78,79,80]. A recent study has proposed that CL is also crucial for the association between Bax dimers to form high-order oligomers. Indeed, without the presence of CL, Bax dimers assemble into an inactive oligomer that is not able to form stable pores [81]. Recently, it has also been shown that GSDMs are recruited to different cellular membranes by specific lipids. The recruitment of GSDMD to the PM is mediated by negatively charged lipids, such as PS and phosphatidylinositol phosphate lipids (PIPs) [57], like for MLKL, where the membrane interaction is promoted by the positively charged residues in the 4HB domain [82]. However, GSDMD also permeabilizes CL-rich membranes, suggesting a role for GSDMD in MOMP and in the permeabilization of bacterial membranes, both enriched with this lipid [57,83,84]. 

Lipids affect protein recruitment and pore formation not only through direct protein interaction, but also by affecting the overall membrane properties. Local curvature, lipid organization, membrane density, and fluidity, which are all dependent on lipid composition, may all stimulate PFP binding and membrane insertion [85]. This is the case for lysenin, which binds only to clustered SM, and EqtII, which was shown to bind preferentially to SM at the liquid-ordered phase border [75,86].

In turn, PFPs may induce membrane perturbations beyond pore formation, altering the local lipid environment and membrane organization. This is the case for proapoptotic Bid and Bax, which are able to distinctly remodel CL-containing membranes and stabilize their curvature. In particular, both Bid and oligomeric Bax act by lowering the membrane line tension, thus favoring the opening of the pore [87,88]. Similar behavior has been suggested for actinoporins, which are able to bind to and reshape SM-cholesterol liquid order domains [75]. Finally, the activity of the CDC family member PLY is not limited to pore-driven membrane permeabilization but also involves membrane blebbing, fusion, and aggregation. Specifically, during its oligomerization and pore formation, PLY induces redistribution of lipid components in the bilayer, destabilizing the membrane and generating small vesicles [89].

### 3.2. Assembly of PFPs at the Membrane

Following membrane targeting, the process of pore formation is characterized by protein assembly, which comprises two steps: membrane insertion and oligomerization. Thus far, there are two models proposed to describe the mechanism of membrane insertion by PFPs. In the “concerted” model, after membrane targeting, proteins first oligomerize on top of the membrane to form a ring or “pre-pore” at the membrane interface, and after oligomerization, all units concertedly undergo a conformational change allowing membrane insertion (Figure 1B). This mechanism of pore formation has been observed for many CDC pores, such as Perfringolysin O (PFO) [16], PLY [47], and LLO [90]. In general, CDCs bind to and oligomerize on the membrane surface into a pre-pore of 20–50 monomers. After pre-pore assembly, two small clusters of α-helices (TMH1 and TMH2—transmembrane helical regions 1 and 2) in each monomer unwind and insert into the membrane, forming the final β-barrel [91]. In the second, or non-concerted, model, proteins first undergo conformational changes to insert into the membrane and only then oligomerize and form the pore (Figure 1B). This is the case for the actinoporin EqtII [10] and Bax, which insert into the membrane as monomers, and following extensive conformational changes, quickly self-assemble into more stable symmetric dimers [32,52].

Besides membrane insertion, the oligomerization step can also follow different models. In the sequential model, assembly occurs via the addition of units with the same stoichiometry (Figure 1C), as suggested for the actinoporin EqtII [10] and Bax, in which, after the formation of the stable dimer on the membrane, further oligomerization proceeds by sequential addition of dimeric units [32]. In contrast, in the “non-sequential” model, the assembly occurs through the addition of units with a random stoichiometry, as proposed for the pore-forming toxin ClyA [92] (Figure 1C).

### 3.3. Pore Formation and Structure

PFPs also differ in the type of pore they form. Pores are classified as protein-lined when they are exclusively formed by proteins and as protein-lipid or toroidal pores when both proteins and lipids participate in the pore structure (Figure 1D,E) [93]. In protein-lined pores, protein units inserted into the lipid bilayer form lateral protein-protein interactions that stabilize the pore structure (Figure 1D). Proteins completely span the membrane and expose the hydrophobic regions towards it, while the hydrophilic regions face the lumen of the created water channel. Examples of protein-lined pores are the ones formed by members of the CDC [91] and GSDM [36] families, as well as ClyA [92]. In toroidal pores, lipids participate together with proteins to form the pore structure by directly covering part of the pore edge (Figure 1E). In this type of pore, the membrane curves to form a torus-like channel, where lipids rearrange into a non-bilayer structure, forming a continuum of the two leaflets in order to avoid the exposure of their hydrophobic acyl chains to the aqueous environment. Toroidal pores have been proposed for Bax\Bak and colicins [1,94]. A third class of pores, defined as hybrid pores, has been described for the octameric actinoporin FraC, in which both proteins and lipids contribute to the pore. FraC pore formation, beyond protein-protein interaction, occurs thanks to lipids that do not act only in protein membrane recruitment but also participate in the assembly as a structural element of the pore, where each protein molecule is associated with three molecules of lipids [12,93]. Moreover, for some PFPs, besides ring-shaped pores, the formation of intermediate structures, heterogeneous in size and shapes, such as arc- and slit-like structures, have been reported [6]. In particular, arc structures are characterized by the incomplete presence of the proteins at the pore rim, while the other part is occupied by lipids. This is the case for the apoptotic proteins Bax and Bak that present heterogeneous structures, such as lines and functional arcs and rings [32,33,34,54,55] and for many members of the CDC and related families. The CDC member LLO, for example, oligomerizes in arc-shaped structures that insert into the membrane and form functional pores. These arcs can subsequently fuse into larger assemblies and form rings of circular or irregular shapes and various sizes [47,48]. However, whether arcs represent only intermediate structures that evolve into rings with the addition of new protein units or are stable functional entities is not completely understood yet [55,93,95,96]. 

## 4. Importance and Challenges of Studying Pore Formation

Although PFPs have evolved a common general mechanism of pore formation, we have discussed in the previous sections that different PFPs lead to different oligomer assemblies and pore architectures. The great diversity of PFPs and pores in nature raises the question of why there are so many ways to permeabilize cell membranes. A clear answer to this question is the need for different pores to allow the passage of different molecules based on pore properties such as size and selectivity, as well as their cellular localization. Although cell death due to osmotic imbalance is the most obvious consequence of membrane permeabilization by PFPs, pore formation does not necessarily lead to cell death. There is growing evidence of the importance of pore formation for the passage of signaling molecules to initiate important downstream metabolic pathways, such as inflammatory processes initiated by the release of cytokines through GSDM pores at the PM (see Section 2.2). Moreover, the fatal consequences of pore formation by a particular PFP are also directly related to the extent of injury, namely, pore stability, the number of pores on the membrane, and pore size.

The specific pore architecture and its properties result from the intrinsic properties of the protein and can, in turn, be modulated by interactions with lipids and other proteins in the membrane environment. For example, protein-lined pores usually form well-defined and stable pores due to the stabilization of a β-barrel arrangement via protein-protein interactions. In contrast, toroidal pores are structurally less defined in size and shape due to the flexibility provided by the presence of lipids at the pore edge. The same applies to intermediate structures such as arcs (identified for both α- and β-PFPs), where part of the pore is covered by lipids. The overall picture shows a complex scenario with heterogeneous structures resulting from the degree of stability of the final pores, the presence of intermediate structures, and the contribution of external factors (including lipids) that may modulate pore formation. Furthermore, the process of pore formation is the result of an oligomerization process that can be very dynamic and transient and, as such, is difficult to characterize under steady-state conditions.

Therefore, unraveling the mechanism of pore formation is often challenging as this requires a controlled membrane environment as well as investigative and analytical tools that provide individual, rather than ensemble, information to allow observation of the complex spatial-temporal heterogeneity of PFP structures. In the following sections, we describe how the use of mimetic systems can provide such a controlled environment for the analysis of PFPs (Section 5) and how the advent of single-molecule techniques has overcome the previous limitations in the investigation of pore formation by providing information at the level of the individual protein complex (Section 6).

## 5. Artificial Membrane Systems used to Study Pore Formation by PFPs

**Figure 2:**
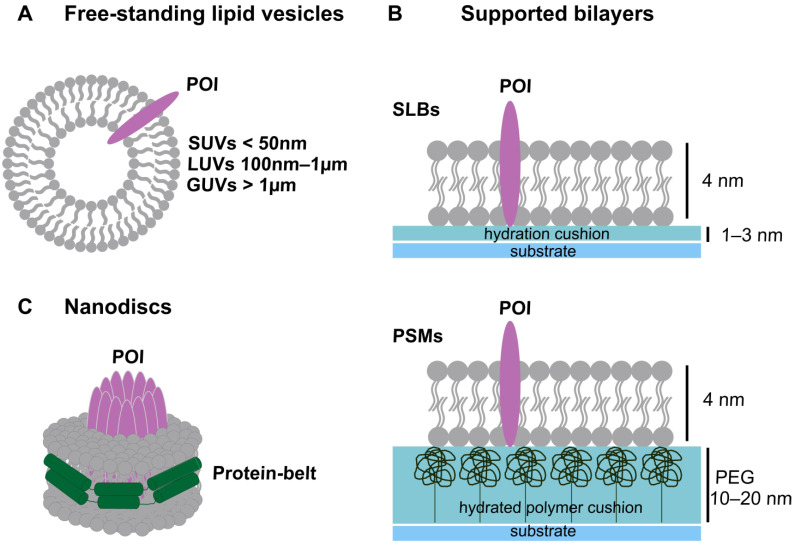
Artificial membrane systems. (**A**) Schematic representation of a free-standing lipid vesicle where a protein of interest (POI) is embedded in the membrane. (**B**) Schematic representation of (**top**) supported lipid bilayers (SLBs), and (**bottom**) polymer-supported membranes (PSMs) with embedded POI. (**C**) Schematic representation of a nanodisc with POIs incorporated in the lipid environment. Lipids are represented in gray and proteins in pink.

Biological membranes play fundamental roles in protecting cells from the environment and enabling the exchange of substances, cell adhesion, transport, metabolism, and cell signaling. To accomplish these multiple functions, biological membranes comprise a complex network of proteins embedded in a lipid bilayer composed of thousands of different lipid species. This high complexity and component variability do not allow for dissecting the individual contribution of proteins and lipids to cell function. Model membranes allow for overcoming this limitation by providing minimal membrane systems in which individual components can be introduced and monitored to identify their relevance to the functional activity of PFPs [97,98,99]. The advantages of using model membranes are manifold. On the one hand, they offer the possibility to study the contribution of individual lipids (lipid charge, geometry, as well as length and saturation of the fatty acyl chains) to protein function and membrane properties, such as membrane curvature and fluidity. On the other hand, the reconstitution of purified proteins into lipids allows for focusing on selected interactions and functions. Free-standing lipid vesicles, formed by spherical assemblies of lipid bilayers that enclose an aqueous compartment, are commonly used model membranes, and they are classified by size: small unilamellar vesicles (SUVs), which are less than 50 nm in size, large unilamellar vesicles (LUVs), which are between 100 nm and 1 µm in size, and giant unilamellar vesicles (GUVs), which are larger than 1 µm (Figure 2A). SUVs and LUVs can be generated via the breakage of multilamellar liposomes using different physical methods, including extrusion, sonication/ultrasonication, or detergent dialysis and homogenization [99,100]. GUVs are mostly obtained using the electroformation method [101,102]. Thanks to their free-standing nature, soft exterior, geometry (curvature), and size, these vesicles are good models to study the membrane permeabilization processes of PFPs and are distinctly employed to mimic cellular compartments of a similar size. A big advantage of GUVs is that they can be easily visualized via optical microscopy and have a size comparable to that of eukaryotic cells, which is useful to recapitulate PM properties (e.g. curvature) [103,104,105]. These vesicles are used to test protein activity in binding and permeabilization assays (as explained in Section 6).

Supported lipid bilayers (SLBs) are another extensively used membrane system characterized by flat membranes formed on planar surfaces with a thin liquid layer between the bilayer and the surface (Figure 2B) [106]. SLBs are formed on hydrophilic and smooth surfaces, such as glass, mica, or silica, due to the ability of these materials to facilitate spontaneous bilayer formation through vesicle adsorption and subsequent fusion driven by calcium ions [107,108,109]. Indeed, a big advantage of SLBs over free-standing vesicles is their planar configuration, which removes membrane curvature contribution and makes them ideal systems for several biophysical techniques, such as surface plasmon resonance (SPR), atomic force microscopy (AFM), and total internal reflection fluorescent (TIRF) microscopy. SLBs have been extensively used to study the assembly of PFP complexes through AFM [95,110,111] and single-molecule TIRF microscopy [32,54]. However, a big limitation of SLBs is that, when incorporating transmembrane proteins, the hydration cushion between the support and the bilayer may not be sufficient to prevent protein interaction with the supporting surface, causing protein fixation and also potentially protein deformation and denaturation. To overcome this limitation, a polymeric layer can be introduced as a spacer between the substrate and the lipid bilayer to form polymer-supported membranes (PSMs) that allow protein diffusion into the bilayer and real-time tracking of protein assembly (Figure 2B) [112,113,114,115]. PSMs have been used to study cholera toxin subunit B redistribution towards liquid-ordered domains in the membrane [116] and to identify membrane pore formation by ɑ-hemolysin in a membrane biosensor created by generating a membrane bilayer on a conducting polymer [117].

Finally, nanodiscs (Figure 2C) are soluble discoidal phospholipid bilayers surrounded by a protein belt. They can include integral membrane proteins and they represent a useful platform for functional and structural studies [118,119]. They are homogeneous in size and have high stability in water solutions, providing access to both sides of the lipid bilayer. These characteristics make them ideal tools for protein structural studies using AFM, nuclear magnetic resonance (NMR), and cryo-electron microscopy (cryo-EM). Nanodiscs are assembled by solubilizing lipids, the belt-forming protein, and the membrane protein of interest (POI) with detergents, followed by detergent removal. The size of the nanodisc is determined by the type of protein used to create the belt, and it can be tuned appropriately to allow the accommodation of the complex of interest. In particular, membrane scaffold proteins (MSPs) are able to form nanodiscs in the range of 8–18 nm, while newly developed NW (nanodisc-width) proteins such as NW30 and NW50 can produce circularized nanodiscs 30–50 nm in diameter [120,121,122]. Recently, lipid-polymer nanodiscs, such as styrene\maleic acid lipid particles (SMALPs) and diisobutylene-maleic acid lipid particles (DIBMALPs), with a size ranging from 5 to 100 nm, have been developed [123,124,125,126,127]. Compared to classic nanodiscs, they do not require detergents to form and offer the advantage to extract membrane proteins together with the surrounding lipids directly from cellular membranes [127,128]. 

Model membrane systems are invaluable tools for the mechanistic and structural analysis of PFPs. However, it should be kept in mind that they provide a simplified scenario of cellular processes; therefore, they should be considered to complement, not substitute, measurements of protein complexes in their physiological cell environment.

## 6. Methods for the Structural Analysis of PFP Complexes at the Single-Molecule Level

### 6.1. Pore Assembly by Single-Molecule Imaging (SMI) Fluorescence Microscopy

After the initial binding of PFPs to the membrane, deep conformational changes allow for membrane insertion and oligomerization. However, for the majority of PFPs, the exact molecular mechanisms of pore assembly have remained unclear [129]. This is mainly due to the highly transient nature of the oligomerization process, which may include short-living intermediates. 

In the past, classical biochemical methods, such as cross-linking, gel filtration, native gel, and analytical ultracentrifugation (AUC) [130,131], have been used for determining the oligomeric state of PFPs [132,133,134]. However, these techniques only provide information on the oligomerization process in steady-state conditions. To overcome these issues, fluorescence techniques such as Förster resonance energy transfer (FRET) and fluorescence correlation spectroscopy (FCS) have been introduced to monitor the real-time interaction of proteins in the process of oligomerization. However, all these techniques provide ensemble information on the stoichiometry populations, which does not take into account the heterogeneity of the oligomerization process.

In the last few decades, the development of single-molecule imaging (SMI) microscopy has overcome these limitations, allowing to reveal the spatio-temporal nanoscopic details of protein oligomerization at the single-molecule level. Single-molecule-based approaches, such as subunit stoichiometry determination via TIRF microscopy, single-molecule FRET, both based on fluorescence microscopy, and mass photometry, which does not require the presence of fluorophores (discussed in the next section), have become useful tools for the investigation of oligomerization in the membrane environment [135]. SMI based on fluorescence microscopy is usually performed in combination with TIRF microscopy, which allows single-particle detection in artificial or plasma membranes by illuminating only a thin plane of the sample in order to strongly reduce background fluorescence [136,137]. In SMI experiments, protein concentration plays a fundamental role, as this technique requires low protein density to allow for single-molecule detection. However, this can be a limitation either because protein concentration cannot always be modulated (as for some proteins in the cell membranes) or because the low density affects the kinetics of the assembly. 

**Figure 3:**
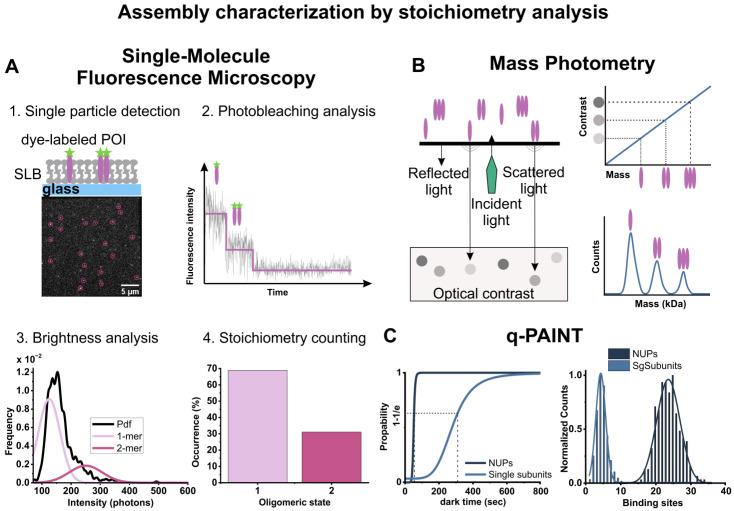
Techniques used to determine pore assembly via stoichiometric analysis. (**A**) 1: schematic representation of dye-labeled POI assemblies reconstituted in SLBs (**top**) and a TIRF representative image of fluorescently labeled protein complexes (bright spots, **bottom**). Stoichiometric analysis via SM fluorescence microscopy involves the detection of single particles at the membrane surface (circles in the image); 2–4: two analysis approaches (step-wise photobleaching and brightness analysis) can be used to determine the percentage of occurrence of the oligomeric species (see text for details). (**B**) Principle of mass photometry (**left**). MP detects the change in reflectivity caused by interference of reflected light and scattered light by biomolecules landing on a glass microscopy slide. Particle landing events appear as diffraction-limited events and the contrast of each event is proportional to the mass of the landed biomolecule (**upper right**). Recording the contrast of many events results in a distribution of the species in solution. After calibration with molecular weight standards, the light-scattering signals of single biomolecules can be converted into MWs and represented as an MW distribution (**bottom right**). (**C**) Stoichiometry qPAINT analysis of NUPs forming the nuclear pore complex. qPAINT enables the signal frequency to determine the number of binding sites; a high number of tagged molecules per structure results in an increased binding frequency of the imager strand and thus leads to a reduced dark time. Comparing the mean dark time to the dark time of a stoichiometrically known sample, used for calibration, allows the determination of the unknown number of subunits. (**Left**): signal frequencies as mean dark times are shown for single subunits and complete NUPs. (**Right**): distribution of binding sites for complete NUPs (dark blue) and single subunits (light blue).

Subunit stoichiometry determination allows for unraveling the distribution of different oligomeric species, giving it a significant advantage over Western blotting, cross-linking, or chromatographic methods that provide ensemble readouts [136,138]. In performing subunit stoichiometry analysis using TIRF microscopy, the recombinant POI is labeled with a fluorophore of choice in a ratio of 1:1, and the protein complex is reconstituted into membrane systems such as SLBs (Figure 3A). At the right protein concentration, complex particles are visualized as diffraction-limited isolated spots. Subunit stoichiometry counting can be achieved using two methods: step-wise photobleaching and brightness analysis (Figure 3A). In the step-wise photobleaching approach, the step-wise decrease of the protein complex intensity is monitored over time until complete bleaching. Since each photobleaching step corresponds to the bleaching of one fluorophore, by counting the number of steps, it is possible to determine the number of subunits in the complex [139,140]. This approach, initially developed by Ulbrich and Isacoff to measure the subunit stoichiometry of GFP-tagged NMDA receptors [139], has been applied as well for characterizing calcium channels [141] and, in the context of PFPs, EqtII and Bax oligomerization [32,142]. A major disadvantage of this method is that it is generally limited to evaluating protein complexes with low-number stoichiometry (up to 4–5 subunits) due to the increased noise in detecting photobleaching steps associated with a higher number of fluorophores [143]. Furthermore, this approach requires monitoring the intensity of the particle over time, and therefore the analysis of diffusing proteins should be complemented by tracking algorithms. In the brightness analysis, protein stoichiometry is directly calculated from the fluorescence intensity of the individual particles [144,145]. Specifically, the brightness of each particle is compared to the brightness of a single monomer used for calibration. Monomers with a single bleaching step are selected by the photobleaching approach, and their intensity value is used to calculate a Gaussian distribution model of the mean intensities corresponding to higher-order oligomeric species [136,145]. This approach has been successfully used to determine the stoichiometry of oligomeric complexes of PFPs in cells and model membranes [10,32,54]. In this way, the oligomerization mechanism of Bax and Bak in SLBs has been revealed, showing that Bax initially binds to the membrane in a monomeric state followed by fast self-assembly, which leads to the formation of multiple different species all based on dimer units [32]. Oligomerization of the other apoptotic executor Bak still proceeds via dimer assembly; however, it is less affected by protein concentration, suggesting a mechanistic difference in the assembly of these two proteins [54]. The same method has also been successfully used to analyze the stoichiometry of actinoporin EqtII complexes at the PM of living cells, showing that the protein does not organize into a unique oligomeric form but it exists as a mixture of oligomeric species including monomers, dimers, tetramers, and hexamers [10]. The brightness analysis allows for resolving higher-order oligomers compared to the step-wise photobleaching approach. However, this method strictly relies on the quality of the calibration measurement, which may vary from experiment to experiment due to manifold factors related to bilayer preparation, the quality of the monomers, and variations in the microscope settings and performance, such as inhomogeneous illumination over the imaging area. The advantages of this method are that it is less sensitive to noise compared to the step-wise photobleaching method and it is versatile as it allows for measuring the stoichiometry of not only immobile but also mobile proteins diffusing in the membrane [144]. Both stoichiometry analysis methods involve a quite laborious process of post-imaging data processing, considering that reliable experiments are based on the analysis of several thousand particles. To overcome these limitations and speed up data analysis, recently, algorithms based on deep learning neural networks have been implemented for the automatic analysis of step-wise photobleaching [146] and brightness analysis [147]. 

Single-molecule FRET (smFRET) is another powerful technique with single-molecule sensitivity that has the unique advantage of the ability to measure intra- and inter-molecular distances in real time with high spatial precision, thus being particularly advantageous in characterizing short-lived intermediates. smFRET has been used to determine the monomer-to-protomer conversion of ClyA in the presence of detergent, which triggers a conformational change in the monomer and the formation of an intermediate assembly that further oligomerizes to form the final pore [92,148]. smFRET has also been employed to study conformational changes in LLO in GUVs and their influence on lipid organization during membrane permeabilization [149].

### 6.2. Mass Photometry

Mass photometry (MP) is an in vitro label-free optical method that was recently developed for the quantification of the molecular mass of biomolecules at the single-molecule level and the determination of the degree of purity and aggregation of a protein sample [150]. This technique has become increasingly used to quantify protein-protein interactions and extract the oligomeric state of a protein complex because it does not require previous labeling of the protein with a fluorophore [151,152]. MP is based on the interferometric detection of light scattering. The sample is deposited on a glass surface that is illuminated by a laser generating reflected and back-scattered light, which is detected by a camera. Biomolecules landing on the glass surface cause a change in the local refractivity and generate a light-scattered signal that can be used to determine their molecular weight (MW) using appropriate calibration samples (Figure 3B) [153]. MP can accurately measure the molecular mass of biomolecules in the range of 40 kDa–50 MDa. It has a 2% mass accuracy, up to 20 kDa resolution, and 1 kDa precision [153]. It has the relevant advantages of being non-invasive, it requires low sample amounts and minimal sample preparation, and the potential for high-throughput analysis. Conversely, this technique requires working with protein concentrations of up to 100 nM, limiting the accessible Kd range from low pM to a few hundred nM, and it requires purified samples to remove the non-specific background. Data analysis is straightforward but needs a calibration curve based on known MW biomolecules. In the last two years, the applications of MP have been extended to membrane proteins in membrane-mimetic systems. For example, both SMALP- or MSP-based nanodiscs containing the *Escherichia coli* potassium channel KcsA (K channel of streptomyces A) have been characterized using MP, revealing stoichiometric and functional details [154]. Finally, improved strategies for image processing and analysis have recently allowed the extension of MP applications to study the association of membrane-associated proteins on SLBs via label-free tracking [155]. Mass-sensitive particle tracking (MSPT) or dynamic mass photometry (DMP) have been used to determine the turnover and stoichiometry of protein complexes, as in the case of MinD/MinDE of the *Escherichia coli* Min system [156] and the GTPase Dynamin-1 [157], paving the way for their application to other systems, including PFP assembly.

### 6.3. Stoichiometry and Structural Analysis via Super-Resolution Microscopy

Besides protein complex assembly, an understanding of the complex structure is equally important to understanding pore function. Super-resolution microscopy (SRM) techniques, such as STORM, PALM and FPALM, STED, MINFLUX, and DNA-PAINT, have become valuable tools for providing structural information of PFP complexes at the single-molecule level. Although structural techniques such as X-ray crystallography and cryo-EM provide resolution at the atomic level, SRM offers the great advantage of a spatial resolution of up to a few nanometers in measurements taken directly in the preserved native cellular environment. Furthermore, unlike other structural techniques, SRM does not require highly homogenous samples and provides relevant information in terms of sample heterogeneity, such as the size, shape, and spatial organization of protein complexes [158,159]. Compared to the other SRM techniques, STED has the ability to provide time-resolved dynamics information on pore complex assembly and membrane reorganization in the presence of PFPs. STED has been applied to monitor lipid dynamics and membrane rearrangements in cholesterol-rich model membranes in the presence of the PFT LLO, mimicking the membrane reorganization that occurs after invasion by *Listeria monocytogenes* [160,161]. Finally, multicolor STED has been used to visualize Bax and/or Bak structures on the mitochondria of apoptotic cells [54].

**Figure 4:**
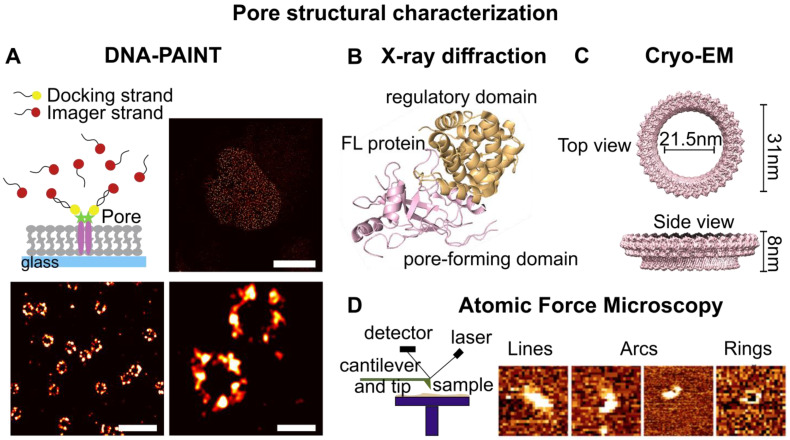
Techniques for the structural characterization of membrane pores. (**A**) DNA-PAINT schematic representation and analysis of the nuclear pore complex revealing the ring-shaped structure of the pores. Scale bars: 10 µm for the overview (**top right**), 500 nm (**bottom left**), and 100 nm (**bottom right**) for the zoomed-in images. (**B**,**C**) X-ray crystallography and cryo-electron microscopy allow for determining the structure of a protein at atomic resolution. (**B**) Crystal structure of FL GSDMD obtained via X-ray diffraction (pdb: 6N9O). (**C**) Top and side views of GSDMD pore structure obtained via cryo-EM revealing a full pore of 33 subunits (pdb: 6VFE). (**D**) Atomic force microscopy schematic representation (**left**) and AFM images of Bax structures on model membranes (picture size 100 nm) (**right**, adapted from [54]).

More recently, DNA PAINT has emerged as a powerful approach for the structural characterization of membrane assemblies [162]. DNA-PAINT is a localization SRM method based on the transient binding of short dye-labeled oligonucleotides (imager strands) to their complementary target (docking) strands. The docking strand is generally fused to an antibody or smaller labeling agents, such as aptamers or nanobodies, that are able to target the POI (Figure 4A). The binding and unbinding of the imager strand to the docking strand generates the blinking events necessary to enable stochastic super-resolution microscopy. Because of the large pool of existing fluorophores in solution, photobleaching during DNA-PAINT is negligible, thus allowing to collect a large number of blinking events and achieve a spatial resolution of down to a few nanometers [163]. An extended application of DNA-PAINT is quantitative PAINT (qPAINT), which allows a stoichiometric quantification of molecules within nanoscale assemblies (Figure 3C) [164]. The DNA-PAINT and qPAINT approaches have already been applied to determine the pore structure and the number of subunits of the nucleoporin (Nup) assembly forming the nuclear pore complex (NPC) [164,165].

### 6.4. Structural Characterization at Atomic Resolution

X-ray crystallography and cryo-EM microscopy are structural techniques with atomic resolution extensively used for structural studies of protein and protein complexes. In X-ray crystallography, high-resolution protein structures are determined by analyzing the X-ray diffraction pattern obtained from a protein crystal. This technique can be successfully applied to any soluble protein that can be crystallized in high amounts, and it is useful to study protein-ligand interactions [166]. The structures of several PFPs in their inactive soluble form have been determined using X-ray crystallography, elucidating critical domains and residues for protein activity. For example, the crystal structure of the full-length GSDMD has revealed the mechanism of auto-inhibition of the inactive full-length protein (Figure 4B) [167]. Furthermore, the structure of GSDMD in complexes with inflammatory caspases has elucidated the residues involved in their interaction and GSDMD activation [168,169]. However, generally, crystallization can be difficult for biomolecules with a high molecular weight. This, together with the requirement of high stability of the purified protein in solution, hinders the application of this technique to membrane proteins and protein complexes.

Transmission electron microscopy (TEM) and cryo-EM have now become the techniques of choice to determine the atomic structure of protein complexes in a lipid environment. In cryo-EM, samples are prepared through cryopreservation and, unlike in single-crystal X-ray diffraction, the rapid-freeze treatment of the sample maintains the proteins in a form close to their native state. In addition, this method requires only a small amount of sample (~0.1 mg) and does not need protein crystallization. Importantly, membrane proteins, which are hard to purify and study in solutions, can be extracted with detergents and polymers from their natural environment or be imaged in their lipid environment, for example, by the use of nanodiscs (Figure 2C). The main drawback of this technique is that the particles are detected in unknown orientations. Hence, structure determination of biological macromolecules via cryo-EM is limited to large complexes or low-resolution models. Cryo-EM has permitted the structural arrangements of large membrane pores formed by PFPs, such as GSDMA3 and GSDMD, to be studied. The cryo-EM structure of the GSDMA3 pore revealed a pore size of 18 nm assembled by 27 units, while GSDMD forms pores of 21 nm in diameter consisting of 33 units (Figure 4C) [35,36]. The pore structures highlight that GSDMs undergo deep conformational changes in the active N-domain during membrane insertion, and reveal important protein-lipid and protein-protein interaction patches involved in pore formation. In detail, each pore-forming subunit presents a “hand-like” structure with two inserted β-hairpins, the “fingers”, and a globular domain, “the palm”, which forms the rim of the pore. Furthermore, the pore structure of GSDMD discloses the presence of acidic patches in the pore conduit that influence cargo transport and explain its selectivity towards mature cytokines.

### 6.5. Determination of Pore Structure and Functionality via Atomic Force Microscopy (AFM)

AFM is a powerful non-optical, scanning-based technique that provides nanoscopic-resolved 3D images of biological samples. This is achieved via the use of a very sharp tip (generally in the range of 1–20 nm in diameter) that scans the sample surface and moves in response to tip–surface interactions. Because it provides a direct, real-time three-dimensional image of the sample, AFM offers the unique advantage of imaging PFP structures and directly correlating them with their ability to create functional pores in the membrane [96,170]. Several ring- and arc-shaped PFP structures have been visualized using AFM, including MACPF\CDC [18,30,171,172], GSDMs [95,110], and Bax and Bak [33,54,88,173], in SLBs (Figure 4D). Recently, AFM studies have also provided an understanding of bactericidal killing by the membrane attack complex (MAC) pores by imaging pore formation on live cells [174,175], as well as of the molecular mechanisms forming the basis of lipid specificity by perforins [76]. During the last few years, progress in AFM has led to the development of high-speed AFM, which can be used to monitor the real-time assembly of proteins in membranes. This approach has been used to monitor the dynamic assembly of several PFPs pores in SLBs, such as LLO, lysenin, and perforin, revealing pore formation at different steps of oligomerization and the overtime conversion of arcs into stable ring-shaped oligomers [176].

### 6.6. Molecular Dynamics Simulations

In recent years, computer simulations of complex biomolecular systems have become an important complementary tool for understanding the atomistic details, interactions, and complex properties of PFPs. They help explain experimental data and enable the investigation of time scales that are difficult to access with common experimental techniques. Furthermore, computer simulation also has predictive power, thus aiding in the design of new experiments. Atomistic simulations have been used to model protein-lipid interactions critical for membrane binding and oligomerization of PFPs. For example, all-atom molecular dynamics simulations have been used to determine the molecular details of membrane reorganization by the CDC family member LLO. Simulations were performed by considering two distinct states of the oligomerized protein: a membrane-bound state and a membrane-inserted state. In the membrane-bound state, four D4 subunits formed a pre-pore on the membrane surface, while the membrane-inserted state presented an arc-shaped tetrameric assembly. Moreover, the simulations were able to identify lipid mobility and an increase in local density in cholesterol next to the putative cholesterol-interacting subunits of the membrane-bound state, confirming the role of these domains in CDCs [177]. The stabilizing role of cholesterol for this protein family also has been confirmed with MD simulations of ClyA [178]. Furthermore, the development of coarse-grained molecular dynamics (CGMD), which allows investigation over longer time scales, has provided further relevant insights into protein folding, protein-protein and protein-lipid interactions, and into the mechanism of assembly of larger oligomeric complexes. For example, CGMD simulations have been used to establish key steps of PLY pore formation, confirming critical membrane-binding sites identified via experimental mutagenesis [179] and to determine the role of lipids and salt in modulating the electrostatic filtering of GSDMD pores against cytokine precursors [180].

## 7. Methods for Assessing Pore Functionality

**Figure 5:**
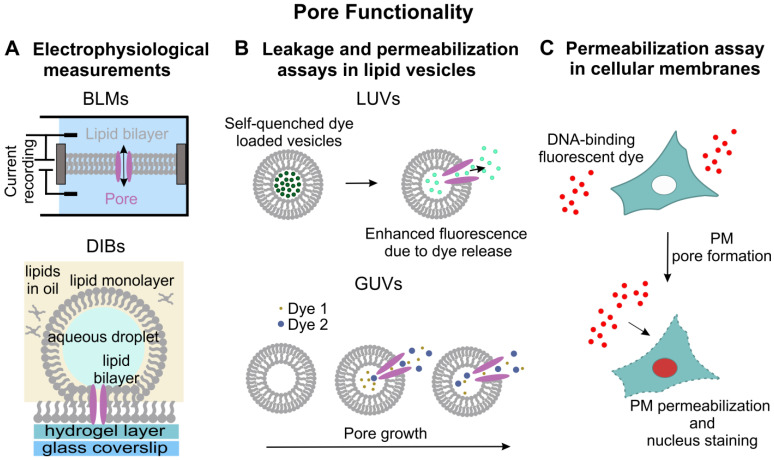
Methods used to evaluate pore functionality. (**A**) Electrophysiological measurements of currents generated by pore formation can be detected in different membrane model systems, such as planar membranes (**top**) or DIBs (**bottom**). (**B**) Free-standing vesicles are used in permeabilization assays with recombinant proteins (see text for details). (**C**) In cells, membrane-impermeant dyes binding to DNA are used as markers of membrane permeabilization of the PM.

Pore functionality, namely, the ability of a membrane-inserted protein complex to perforate the membrane and allow the passage of molecules, is an essential feature of pore formation. Functionality assessments can be achieved using several methods, and one of the most commonly used is electrophysiology. In vitro electrophysiology is based on the use of black lipid membrane (BLMs) systems that are planar bilayers covering a hole within a non-conducting polymer sheet (such as Teflon), separating two electrolyte solutions (Figure 5A). PFPs can be reconstituted in this bilayer and their electrophysiology probed in different physicochemical conditions or with different substrates or modulators [181,182]. These approaches have been used for the real-time detection of individual membrane pores, for example, in the context of studying the conductance, size, stability, ion selectivity, and dynamics of pore opening and closing events of bacterial PFPs [183,184]. More recently, ionic current measurements have been integrated with single-molecule fluorescence imaging on droplet interface bilayers (DIBs). DIBs are formed by the deposition of an agarose hydrogel layer on the surface of a microscope coverslip, followed by the deposition of a phospholipid-in-oil solution and finally an aqueous droplet. The lipid molecules self-arrange in monolayers and then, under gravity, form a bilayer in contact with the hydrogel. Electrodes can be applied to both sides of the membrane and electrophysiological studies can be performed (Figure 5A). These measurements can be easily combined with microscopy analysis in the presence of fluorescence-labeled proteins to examine protein assembly [185], pore formation [186], and lipid dependence [187], as in the case of the single-molecule study of assembly and membrane insertion of PFO [188]. Electrophysiology combined with fluorescence biosensing has been used in a free-standing membrane bilayer to display GSDMD pore dynamics in vitro [189]. 

Other assays used to test pore functionality include ensemble permeabilization measurements using liposome leakage assays. These are based on the use of vesicles, generally LUVs, loaded with a fluorescent dye [183] or a self-quenching dye, such as calcein, carboxyfluorescein [190,191], or the fluorescent dye/quencher pair 8-aminonaphtalene-1,3,6-trisulfonic acid (ANTS) and p-xylene-bis-pyridinium bromide (DPX) [192]. After disruption of membrane integrity by PFPs, subsequent leakage results in measurable fluorescence due to the dequenching of the dye (Figure 5B). Assay variations use liposomes incorporated with terbium ions (Tb^3+^) and the chelator dipicolinic acid (DPA) provided in the solution, outside the liposomes. Membrane damage by PFPs induces the leakage of Tb^3+^ into the external buffer containing DPA, leading to a fluorogenic reaction between Tb^3+^ and DPA and the formation of the Tb^3+^-DPA complex, which is 10,000 times more fluorescent than the free Tb^3^ [193]. This assay has been used to prove that MLKL forms permeable pores, thereby disrupting cell membrane integrity and causing cell lysis [194]. Permeabilization assays can also be performed at the individual vesicle level with the use of GUVs that can be directly visualized via optical microscopy (Figure 5B). This method not only allows for observing membrane permeabilization via the GUV uptake of fluorescent dyes but also estimating pore stability and pore size by monitoring the overtime uptake of dyes of different sizes (Figure 5B). The size of membrane pores in vesicles can be estimated by measuring the uptake of probes of different sizes, such as fluorescein-labeled dextrans [195]. This method has been used to determine that Bax disrupts liposomes via pore formation and that pores can be of different sizes, allowing the passage of different dyes [196,197]. 

Finally, pore functionality can also be assessed directly in cells via determining cellular uptake of plasma membrane-impermeable dyes [198] (Figure 5C). These assays are performed by adding these cell-impermeant fluorescent dyes to the medium and by monitoring the overtime dye internalization as an indicator of pore formation at the PM [58,199]. Nucleic acid high-affinity dyes such as 7-aminoactinomycin (7-AAD), propidium iodide (PI), and ethidium bromide (EtBr) are commonly used to stain pyroptotic or necroptotic cells that present pores at the PM.

## 8. Conclusions and Outlook

Pore formation has emerged as an ancient cell-killing mechanism used by organisms in manifold physiological and pathological settings. Despite the common ability to perforate cellular membranes, PFPs have different properties, culminating in different methods of targeting specific cellular membranes, different mechanisms of pore formation and different pore functionalities. The recent advances in membrane biophysics methods has provided deep insights into the study of the mechanism of membrane pore formation at the single-molecule level both in artificial membrane systems and in cells. These methods allow sub-nanometric-to-atomic resolution of pore architecture and reveal important details of protein-protein and lipid-protein interactions in the membrane pore. SMI fluorescence microscopy is also used extensively to study the dynamics of pore assembly.

However, efforts still need to be made to link the structural information provided by these techniques to the ability of the assembled protein complex to form functional pores. So far, functional studies have been largely limited to bulk electrophysiological or permeabilization experiments in vitro. An implementation in this direction would make it possible to answer relevant scientific questions. For example, are smaller oligomers equally functionally relevant for the passage of signaling molecules? We are still far from understanding how the different stages of membrane permeabilization affect downstream signaling processes. Uncovering the functional consequences of different PFP oligomer units would explain the presence of heterogeneous pore structures at the membrane. 

Answering these questions is complicated by the short-lived and transient nature of the intermediate oligomeric structures during the process of pore formation, which are either in the form of membrane-associated small oligomers or intermediate assemblies inserted into the membrane. Important steps in this direction have been made by combining SMI and single-channel electrophysiology [200] or single-molecule tracking and single-channel optical recording [188]. Finally, the advent of high-speed AFM has partially filled this technical gap, allowing real-time correlation of oligomer assembly with the formation of membrane holes. Nevertheless, such combined structural–functional characterization is mainly limited to reconstituted membrane systems, and it is difficult to perform in native cellular membranes. 

The molecular investigations of pore assembly by PFPs are also of great relevance for the design of small molecules, nanoparticles, and neutralizing antibodies that hinder pore formation for therapeutic purposes [66,201]. Drugs and therapeutic antibodies designed for PFPs can be used to selectively permeabilize cells for in vitro studies or to build immunotoxins specifically directed against cancer cells [202]. For example, Naptumomab is an immunotoxin developed for the treatment of solid tumors, including renal cell carcinoma. Its therapeutic effect is associated with the action of a bacterial superantigen that activates T cells, the SAg staphylococcal enterotoxin A (SEA) [203]. Moxetumomab has been recently approved against B-cell cancer and it is composed of the pore-forming domain of Pseudomonas exotoxin A and an anti-CD22 antibody, which is responsible for binding to the cell surface receptor CD22 expressed on malignant B cells, thus delivering the toxin and leading to cell death [204,205]. PFPs are also being modified to deliver drugs into mammalian cells [206] in a controlled and specific manner, bypassing the membrane barrier [207]. 

Overall, the continuous development of advanced biophysical methods is paramount to advance our understanding of how membrane pores are regulated and to devise new therapeutic strategies to control the pathological consequences of membrane permeabilization. 

## Figures and Tables

**Table 1:** Classification of PFPs and details on pores and pore formation.

PFPs in Non-Regulated Cell Death						
PFP Family	PFP	Type of Pore	Membrane Receptor	Pore Stoichiometry	Pore Size	References
**Actinoporins**	EquinatoxinII (EqtII)	α-hybrid	Sphingomyelin	4–6	2 nm	[10,11]
	FragaceatoxinC (FraC)	α-hybrid	Sphingomyelin	8–9	11 nm	[12,13,14]
	SticholysinI and II (StnI\II)	α-hybrid	Sphingomyelin	4	11 nm	[15]
**Cholesterol-dependent cytolysins (CDCs)**	PerfringolysinO (PFO)	β-protein-lined	Cholesterol	35–37	34.5–37.5 nm	[16]
	ListeriolysinO (LLO)	β-protein-lined	Cholesterol	variable	variable	[17]
	Suilysin (SLY)	β-protein-lined	Cholesterol	37	31.9 nm	[18]
	Pneumolysin (PLY)	β-protein-lined	Cholesterol	44	26 nm	[19]
**Colicins**	ColicinIA	α-toroidal	Bacterial membrane \ colicin receptor	3	8 nm	[20,21]
	Colicin U	α-toroidal	?	?	1 nm	[22]
**Cytolysin A**	ClyA - HemolysinE	α-protein-lined	Cholesterol	12–14	11–12 nm	[23,24]
**Hemolysins**	α-hemolysin (Hla)	β-protein-lined	Disintegrin\ADAM10	7	10 nm	[25]
	Leukocidin	β-protein-lined	?	4 + 4	?	[26]
**Aerolysins**	Lysenin	β-protein-lined	Sphingomyelin	9	3 nm	[27,28]
**MACPF**	MAC	β-protein-lined	?	22	11 nm	[29]
	Perforin	β-protein-lined	Ca^2+^-Phosphatidylcholine/ Lipid order	20	11 nm	[30,31]
**PFPs in Regulated Cell Death**						
**PFP Family**	**PFP**	**Type of Pore**	**Membrane Receptor**	**Pore Stoichiometry**	**Pore Size**	**References**
**Bcl-2**	BAX\BAK	α-toroidal	Negatively charged lipids	Multiple of dimers	variable	[32,33,34]
**GSDMs**	GSDMA3	β-protein-lined	Negatively charged lipids	27	28 nm	[35]
	GSDMD	β-protein-lined	Negatively charged lipids	33	31 nm	[36]
\	MLKL	?	Negatively charged lipids	?	4 nm	[37]

## Abbreviations

PFPs, pore-forming proteins; PFTs, pore-forming toxins; RCD, regulated cell death; AFM, atomic force microscopy; EM, electron microscopy; SMI, single-molecule imaging; SUVs, small unilamellar vesicles; LUVs, large unilamellar vesicles; GUVs, giant unilamellar vesicles; TIRF, total internal reflection fluorescence; SLBs, supported lipid bilayers; CL, cardiolipin; PS, phosphatidylserine; PIP2, phosphatidylinositol 4,5-bisphosphate; SM, Sphingomyelin; MOMP, mitochondrial outer membrane permeabilization; PM, plasma membrane.
